# Room-Temperature Air-Only Catalytic Oxidation of Indoor Volatile Organic Compounds: Mechanistic Insights and Emerging Catalysts

**DOI:** 10.3390/molecules31061029

**Published:** 2026-03-19

**Authors:** Dan Zhao, Lisheng Zhang, Yibing Chen, Yongqiang Wang, Hui Ding

**Affiliations:** School of Environmental Science and Engineering, Tianjin University, Tianjin 300350, China; zhaodan20210430@163.com (D.Z.); lishengzhang68@163.com (L.Z.); 1024214019@tju.edu.cn (Y.C.); wangyongqiang@tju.edu.cn (Y.W.)

**Keywords:** indoor air quality (IAQ), volatile organic compounds (VOCs), room-temperature catalytic oxidation in air, single-atom catalysts (SACs), reaction mechanism

## Abstract

Driven by global urbanization and increasing emphasis on sustainable building practices, indoor volatile organic compounds (VOCs) have emerged as a major environmental and health challenge. This review specifically focuses on room-temperature air-only catalytic oxidation of representative indoor VOCs under a recently matured and highly application-relevant research direction. Recent advances are systematically summarized, highlighting catalyst design strategies, air-phase reaction mechanisms, and performance of noble metal catalysts (NMCs), transition metal oxides (TMOs), bimetallic synergistic catalysts (BSCs), and single-atom catalysts (SACs). Emphasis is placed on thermodynamic feasibility, reaction kinetics, oxidation behavior of non-formaldehyde VOCs, and mechanistic insights associated with SACs interfacial synergy, which enable efficient O_2_ activation, high selectivity, and operational stability without external oxidants even under high VOC concentrations. This review provides theoretical foundations and technical guidance for VOCs mitigation and supports the advancement of green, low-carbon, and safe indoor air purification strategies worldwide.

## 1. Introduction

Rapid urbanization and industrialization have raised global concerns regarding indoor air quality (IAQ). Room-temperature air-only catalytic oxidation is defined herein as a catalytic process that utilizes ambient air as the sole oxidant at room temperature (20–40 °C) without external energy input (e.g., light or electricity) or additional chemical oxidants (e.g., ozone or hydrogen peroxide). This strategy has emerged as a promising approach for sustainable indoor air purification due to its mild operating conditions and inherent safety advantages. Volatile organic compounds (VOCs), emitted from building materials, furniture, cleaning agents, and various consumer products, represent one of the major classes of indoor air pollutants and pose significant risks to human health and environmental safety [1]. Owing to their high vapor pressure, high volatility, and diverse toxic and irritant effects under ambient conditions, VOCs such as formaldehyde, benzene, toluene, and ethyl acetate are widely detected in indoor environments. Long-term exposure to these compounds can cause a range of health effects, including respiratory irritation and neoplastic diseases from formaldehyde [2], hematologic toxicity and leukemia risk from benzene, nervous renal toxicity and central nervous system effects from toluene [3]. Several VOCs, including formaldehyde, have been classified by the International Agency for Research on Cancer (IARC) as Group 1 carcinogens [4], making them priority targets for indoor environmental management. In response to the adverse impacts of indoor VOCs, international organizations and national regulatory authorities have progressively strengthened IAQ standards and emission limits. Some countries, including Canada, the United States, Belgium, and China [5,6,7,8], have successively issued updated guidelines, directives, and legislation aimed at tightening exposure thresholds and improving ventilation requirements. These regulatory efforts reflect a global consensus on the urgent need for efficient, safe, and sustainable VOC purification technologies to ensure healthy indoor environments in modern buildings.

Conventional VOC control technologies, such as adsorption [9], photocatalysis [10], and bioremediation [11], have shown varying degrees of effectiveness in practical applications. However, these methods often suffer from limited VOCs removal efficiency, incomplete CO_2_ mineralization, and the risk of secondary pollution, which restricts their ability to meet the increasingly stringent requirements of green and healthy building environments. In particular, photocatalytic and advanced oxidation systems often rely on external energy input or strong oxidants, which may raise safety concerns and produce undesirable byproducts. Some catalytic systems employ ozone or hydrogen peroxide as oxidants to enhance oxidation activity. However, these oxidants are associated with toxicity, corrosion, and material degradation, limiting their applicability in indoor settings. In contrast, room-temperature air-only catalytic oxidation has emerged as a promising and sustainable alternative for indoor VOCs removal, providing mild operating conditions, low energy consumption, and high purification efficiency while avoiding the risks associated with strong oxidants. Consequently, it is particularly attractive for green buildings, intelligent residential environments, and low-carbon indoor air purification applications.

The core of room-temperature air-only catalytic oxidation lies in the rational design of highly active, selective, and durable catalysts capable of efficiently degrading VOCs under these mild conditions. A wide variety of catalyst systems have been developed, including noble metal catalysts (NMCs) [12,13], transition metal oxide catalysts (TMOs) [14], bimetallic synergistic catalysts (BSCs) [15,16,17], and single-atom catalysts (SACs) [18]. By regulating surface active oxygen species, oxygen vacancy concentrations, and electronic structures, these catalysts achieve effective VOC degradation at high space velocities while maintaining stable CO_2_ mineralization rates. Recent research trends increasingly emphasize tailoring catalyst structures to the molecular characteristics and physicochemical properties of different VOCs. Among indoor VOCs, formaldehyde, as a representative polar compound, has been the primary focus of research, with its catalytic mechanisms and kinetics being well established. In contrast, the oxidation of non-formaldehyde VOCs, particularly aromatic hydrocarbons and esters, has been less systematically summarized, and their room-temperature air-only reaction mechanisms are still not fully understood.

Distinct from existing reviews, this manuscript provides a comprehensive and integrative perspective on room-temperature air-only catalytic oxidation of indoor VOCs. Specifically, it systematically summarizes the current research status of both formaldehyde (HCHO) and non-formaldehyde VOCs, highlighting catalyst design strategies, reaction kinetics, and performance trends relevant to air-only oxidation. In addition, it presents mechanistic insights, including thermodynamic feasibility and the role of active-site configurations in controlling reaction rates and oxidation depth. The review also identifies technical challenges and future research directions, providing a forward-looking framework for advancing practical and sustainable indoor air purification technologies. By integrating these aspects, the present work offers a holistic understanding of air-only VOC oxidation at room temperature, bridging fundamental research, mechanistic insights, and practical application strategies.

## 2. Current Research Status on Room-Temperature Air-Only Catalytic Oxidation of Formaldehyde

Formaldehyde (HCHO) is one of the most prevalent and hazardous VOCs in indoor environments, primarily originating from plywood, furniture, coatings, and other construction and decorative materials. It is characterized by high chemical activity, toxicity, and long-term off-gassing, with even trace concentrations (<0.1 ppm) posing significant health risks. The WHO has classified HCHO as a Group I human carcinogen [2,19]. The room-temperature air-only catalytic oxidation has arisen as a promising strategy for the complete mineralization of HCHO into CO_2_ and H_2_O at ambient conditions, without external energy input or the formation of ozone byproducts, thereby preventing secondary pollution.

In recent years, extensive efforts have been devoted to the design and evaluation of catalytic systems for room-temperature air-only catalytic oxidation of HCHO. NMCs [20,21,22] and TMOs [23] have been extensively investigated due to their high catalytic performance and well-established preparation protocols. Meanwhile, BSCs [24,25] and SACs [26,27,28] have attracted growing attention because of their prominent activity, selectivity, and metal atomic utilization efficiency. In particular, composite systems combining noble and transition metals exhibit remarkable interfacial synergistic effects and tunable electronic structures, significantly boosting the room-temperature air-only catalytic oxidation efficiency of HCHO degradation. Representative studies are summarized in Table 1.

### 2.1. NMCs

NMCs show exceptional performance in room-temperature air-only catalytic oxidation of HCHO, as a result of their high efficiency in oxygen activation and facile desorption of reaction intermediates. Catalysts such as Pt/Co_3_O_4_ [29], Pt/NiO [30], and Pt/SnOx [31] have achieved HCHO removal efficiencies exceeding 85% and near-complete CO_2_ mineralization under ambient conditions. The catalytic functions of noble metals depend on efficient O_2_ activation, promotion of intermediate transformation, and enhancement of interfacial synergy and nanoconfinement effects. Noble metals, including Pt, Au, and Pd, possess favorable electronic structures [41], which facilitate strong O_2_ adsorption and activation, leading to the formation of highly reactive atomic oxygen or peroxide species. Ultimately, their surface activity promotes the conversion of intermediates [30,42] (e.g., HCOO^−^, DOM), thereby increasing overall mineralization efficiency. Strong metal-support interactions (SMSI) between noble metals and oxide supports (e.g., CeO_2_, NiO) further enhance the redox properties of catalysts via electronic modulation, driving the formation of reactive oxygen species and surface hydroxyl groups. The construction of porous nanoarchitectures [43] enriches HCHO molecules and directs the transformation of reactants, consequently improving catalytic efficiency and stability.

### 2.2. TMOs

TMOs, characterized by rich redox properties and tunable oxygen vacancy structures, have also demonstrated high activity for room-temperature air-only catalytic oxidation of HCHO. Compared with NMCs, TMOs like MnO_2_ [32] and NiCo_2_O_4_ [33] offer advantages including low cost, environmental friendliness, and abundant availability. Moreover, the diverse surface active oxygen species and variable valence states [44,45] of these catalysts contribute to their considerable activity. Some MnOx-based catalysts [34,35] retain HCHO removal efficiencies above 95% even under high gas hourly space velocities (200,000–300,000 mL·g^−1^·h^−1^) and elevated relative humidity (≥50%), indicating strong resistance to high load and moisture. However, CO_2_ mineralization rates of TMOs are generally lower than those of NMCs. As shown in Table 1, representative NMCs achieve CO_2_ mineralization rates approaching 100%, while TMO catalysts such as MnO_2_/NCNT and δ-MnO_2_ remain below 50%. This lower CO_2_ mineralization may result from intermediate accumulation on the catalyst surface and limited oxygen activation. TMOs operate via lattice oxygen, forming DOM and formate intermediates and leaving surface vacancies. These vacancies and defect-rich sites facilitate O_2_ activation and reactive oxygen species (ROS) generation, mediating conversion of intermediates to CO_2_ and H_2_O. In MnO_2_/NCNT and δ-MnO_2_, the rate-determining steps in intermediate oxidation suggest that limited ROS or active sites may promote intermediate accumulation. To overcome these limitations, researchers need to optimize material composition and interfacial structures to improve overall catalytic performance.

### 2.3. BSCs

BSCs have been developed by loading noble metals (e.g., Pt, Au) or auxiliary metals (e.g., K) onto TMOs (e.g., NiCo_2_O_4_ [36], MnO_2_ [37] and functionalized supports such as zeolites [38] or layered double hydroxides (LDHs) [39]). Various bimetallic synergistic catalytic systems, typically Pt-Ni and Au-Co, have thus been constructed with remarkable catalytic activity, reaction selectivity, and operational stability. These systems exploit the synergistic interplay between support-mediated surface activity and bimetallic interactions, enabling stable HCHO removal efficiencies above 90% and CO_2_ mineralization rates approaching 100% even under high HCHO concentrations (≥150 ppm). Enhanced performance arises from the integration of the superior O_2_ activation ability of noble metals with the abundant oxygen vacancies and reversible redox properties of TMOs. Effective electronic synergy between bimetallic components is thus achieved, establishing strong interfacial coupling effects at the metal-support interface [46]. This synergy promotes HCHO adsorption, activation, and deep oxidation, achieving both high removal efficiency and CO_2_ selectivity, and positioning BSCs as a key direction for research on room-temperature air-only catalytic oxidation.

### 2.4. SACs

SACs have emerged as a rapidly growing research focus for room-temperature air-only catalytic oxidation of HCHO, owing to their exceptionally high metal atom utilization efficiency, well-defined active site structures, and impressive reaction selectivity. Especially, SAC systems constructed by anchoring noble metal single atoms on supports (e.g., MnOx, TiO_2_) [26,27,28] have possessed interfacial synergistic enhancement effects similar to those of BSCs. Benefiting from their unique electronic configurations and unsaturated coordination environments, these catalysts can also effectively activate reactive oxygen species, synergistically promoting HCHO adsorption and conversion through cooperation with the support. Meanwhile, metal aggregation is effectively suppressed, resulting in improved catalyst stability and recyclability. For example, Pt-MnOOH/MnO_2_ and Pt/Mn-TiO_2_ achieve over 98% HCHO removal efficiency and more than 85% CO_2_ mineralization at noble metal loadings below 1%, highlighting their distinguished catalytic activity, reaction selectivity, and regeneration capability. With continued advancements of atomic-scale structural characterization and regulation techniques, SACs are expected to find broader applications in engineering-oriented air-only purification systems.

## 3. Current Research Status on Room-Temperature Air-Only Catalytic Oxidation of Non-Formaldehyde VOCs

With advances in room-temperature air-only catalytic oxidation technology, research efforts have expanded to the catalytic degradation of non-formaldehyde VOCs, including typical indoor pollutants like aromatic hydrocarbons, aldehydes, alcohols, and esters. Benzene, toluene, hexanal, and ethyl acetate are widely present in building materials, cleaning products, and furniture. These VOCs are characterized by low odor thresholds, moderate to high toxicity, and high volatility, and prolonged exposure may result in multi-system damage. Additionally, their stable molecular structures, high volatilization points, and widespread distribution at low concentrations significantly increase the challenges of achieving effective catalytic oxidation under room-temperature conditions.

To address the purification requirements of these VOCs, a range of catalytic systems suitable for room-temperature air-only catalytic oxidation of non-formaldehyde VOCs has been developed. Among them, SACs stand out due to their high activity, adaptability to elevated mass-normalized space velocities, extraordinary removal efficiency, and recyclability. For instance, MnO_x_-based SACs [47,48,49] achieved nearly 100% removal efficiency under high mass-normalized space velocities of 300–450 L·g^−1^·h^−1^ and low VOCs concentrations (≤15 ppm), with minimal loss in cyclic regeneration performance after continuous operation for 9.5–14 h. Similarly, the Pd-0.5/MnO@C catalyst [49] exhibited a high removal capacity of ≥360 mg·g^−1^ for target pollutants such as hexanal and pentanal. Such performance was attributed to the high oxidation activity of Pd single atoms, the interfacial synergistic enhancement induced by MnO, and the hydrophobic confinement effect of the porous carbon structure.

The generation of ·OH radicals through H_2_O-assisted O_2_ activation plays a key role in the room-temperature air-only catalytic oxidation of non-formaldehyde VOCs. Ding et al. [50,51,52,53,54] constructed a series of room-temperature catalytic systems covering SACs, atomically dispersed bimetallic catalysts, and NMCs, investigating both their oxidation performance and the ·OH radical-involved reaction mechanisms. These systems achieved 99–100% removal efficiency of various non-formaldehyde VOCs within concentrations ranging from 15.5 to 265.4 ppm. Remarkably, a catalyst with 0.16 wt% Fe supported on N-doped porous carbon (Fe/NPC) was designed with highly active FeN_4_O_2_ sites, which activated O_2_ and H_2_O to generate ·OH radicals, achieving complete benzene removal with a CO_2_ mineralization rate of 81.7% and stable operation for 72 h. This catalyst also proved broad applicability for the removal of toluene, xylene, ethyl acetate, and other VOCs. The superior performance was ascribed to the synergy among atomic-scale FeN_4_O_2_ active sites, a unique three-dimensional porous network structure, high surface hydroxyl density, and excellent redox properties. Density functional theory calculations further elucidated the oxidation pathway of benzene on the Fe/NPC catalyst surface. The Fe-N/O coordination centers facilitate ·OH radical generation and electron transfer, significantly lowering the reaction energy barrier. Benzene undergoes stepwise dehydrogenation via ·OH to form C_6_H_2_*, followed by ring cleavage yielding linear C_6_H_2_OOH*, which subsequently cleaves into C_3_ intermediates and finally undergoes consecutive hydroxylation and dehydrogenation to be fully mineralized to CO_2_ and H_2_O.

In summary, SACs and atomically dispersed multi-metal catalysts exhibit exceptional activity at low temperatures, high mass-normalized space velocity adaptability, and stable recyclability for the treatment of non-formaldehyde VOCs. By constructing highly active sites, introducing support defects, and enhancing interfacial synergistic effects, these catalysts achieve high VOCs removal efficiencies and deep CO_2_ mineralization. Notably, catalysts such as Fe/NPC, Pt/BCN, and Ni/NAC [50,51,52,53,54] were developed, exhibiting impressive performance in removal efficiency, mineralization depth, and operational stability, providing valuable insights for the design of high-performance catalysts and interface modulation strategies. Therefore, continuous optimization of catalyst structures and interface engineering lays a solid foundation for subsequent scale-up and practical applications in indoor air purification systems.

## 4. Thermodynamic, Kinetic, and Mechanistic Insights into VOCs Oxidation Under Ambient Air-Only Conditions

### 4.1. Thermodynamic Analysis

Typical indoor VOCs, including formaldehyde, benzene, toluene, and ethyl acetate, present pronounced thermodynamic spontaneity during air-only catalytic oxidation at room temperature. Based on the Gibbs free energy equation (ΔG = ΔH − TΔS), the standard Gibbs free energy changes for the oxidation of these VOCs at 298.15 K and 100 kPa (gas phase) are strongly negative, indicating that these reactions are thermodynamically favorable under ambient air-only conditions. Taking benzene oxidation as a representative example (C_6_H_6_(g) + 15/2 O_2_(g) → 6CO_2_(g) + 3H_2_O(g)), the standard enthalpy of formation (ΔH) and molar entropy (ΔS) of the relevant species are 82.93 kJ·mol^−1^ and 269.31 J·mol^−1^·K^−1^ for C_6_H_6_(g), 0.00 kJ·mol^−1^ and 205.14 J·mol^−1^·K^−1^ for O_2_(g), −393.51 kJ·mol^−1^ and 213.73 J·mol^−1^·K^−1^ for CO_2_(g), and −241.82 kJ·mol^−1^ and 188.83 J·mol^−1^·K^−1^ for H_2_O(g), with thermodynamic data obtained from the NIST Chemistry WebBook (National Institute of Standards and Technology, 2025). Using these values, the Gibbs free energy change of this reaction at 298.15 K is estimated to be approximately −3182 kJ·mol^−1^. This large negative ΔG value reflects the strongly exothermic nature of the oxidation reaction and the net entropy increase associated with gas-phase product formation, jointly driving the reaction toward complete mineralization. Overall, thermodynamic analysis confirms that the air-only oxidation of typical indoor VOCs is intrinsically spontaneous at room temperature, providing a solid theoretical foundation for room-temperature air-only catalytic oxidation in indoor environments.

### 4.2. Kinetic Analysis

Although the air-only catalytic oxidation of typical indoor VOCs is thermodynamically spontaneous at room temperature, its practical efficiency is primarily constrained by kinetic barriers. In the absence of external energy input or auxiliary oxidants, the overall reaction rate is governed by activation energy and oxygen activation efficiency, making kinetic analysis essential for evaluating catalyst performance under ambient conditions. Room-temperature catalytic oxidation of VOCs generally follows three classical kinetic models: the Mars-van Krevelen (MVK), the Langmuir-Hinshelwood (L-H), and the Eley-Rideal (E-R) mechanisms. Among these models, MVK pathways dominate on TMOs through direct participation of lattice oxygen and vacancy regeneration, whereas L-H mechanisms are more common on NMCs involving adsorbed oxygen species. E-R pathways describe direct reactions between gaseous VOCs and adsorbed oxygen species, relevant to systems with rapid surface reaction rates [55,56]. The applicability of each kinetic model depends on catalyst type and VOC properties, and the overall reaction rates are closely related to activation energies, which are influenced by catalyst structure, oxygen activation efficiency, and reaction pathways.

Representative activation energies for room-temperature air-only catalytic oxidation of formaldehyde have been reported on various catalysts, providing direct kinetic evidence that the reaction rate is governed by the activation barrier. BSCs such as Au-CeO_2_-R [15] exhibit an activation energy as low as 22.0 kJ·mol^−1^, while TMOs exemplified by 3D-MnO_2_ [44] display a slightly higher value (25.2 kJ·mol^−1^ at 50–78 °C). NMCs, such as Pd@TS-1 [22], achieve a very low activation energy of 10.8 kJ·mol^−1^ at room temperature, whereas SACs like Pt_1_/CeO_2_-S [18] present significantly reduced barriers of 14 ± 2 kJ·mol^−1^. These differences in activation energy translate into markedly distinct reaction rates, confirming that NMCs and SACs possess superior kinetic performance for formaldehyde oxidation under ambient air-only conditions. However, kinetic data for non-formaldehyde VOCs under strictly room-temperature air-only conditions remain scarce, which limits direct kinetic evaluation of these systems under ambient conditions.

Nevertheless, low-temperature catalytic oxidation studies conducted at 100–200 °C still provide valuable comparative insights into intrinsic kinetic barriers. Although these activation energies are measured at higher temperatures, they can serve as qualitative indicators for potential catalytic performance and relative activity trends under ambient air-only conditions. Conventional NMCs [57,58,59,60] exhibit relatively high activation energies for benzene and toluene oxidation, ranging 92–122 kJ·mol^−1^ and 73–128 kJ·mol^−1^, respectively, indicating kinetically constrained behavior under low-temperature air-only conditions. In contrast, nanostructured NMCs (e.g., Pt/TiO_2_ [61], Au/meso-Co_3_O_4_ [62]) and BSCs such as MnO_2_-CeO_x_ [63] and Cu-Mn/HTS-1 [55] display substantially lower activation energies (approximately 26–55 kJ·mol^−1^) for benzene, toluene, and ethyl acetate oxidation, reflecting the effectiveness of multi-metal synergy and interfacial engineering in reducing kinetic barriers. These findings provide comparative guidance for evaluating catalyst activity trends, which may inform the design of highly efficient air-only oxidation systems for non-formaldehyde VOCs under ambient conditions.

Overall, air-only catalytic oxidation of VOCs involves a sequence of diffusion, adsorption, surface reaction with active oxygen species, intermediate transformation, and product desorption, all of which are influenced by pollutant characteristics, catalyst structure, and operating conditions. By lowering activation barriers, enhancing oxygen activation, and optimizing reaction pathways, advanced catalysts, particularly noble metal, bimetallic, and single-atom systems, enable effective VOC removal under kinetically challenging ambient conditions. However, conventional kinetic models are often insufficient to fully describe the distinctive reaction pathways of emerging catalysts, especially SACs, whose isolated active sites and strong metal-support interactions. These features enable unconventional oxygen activation modes and reaction mechanisms.

### 4.3. Oxidation Mechanisms of VOCs Under Ambient Air-Only Conditions

#### 4.3.1. Formaldehyde (HCHO) Oxidation Mechanisms

**Figure 1:**
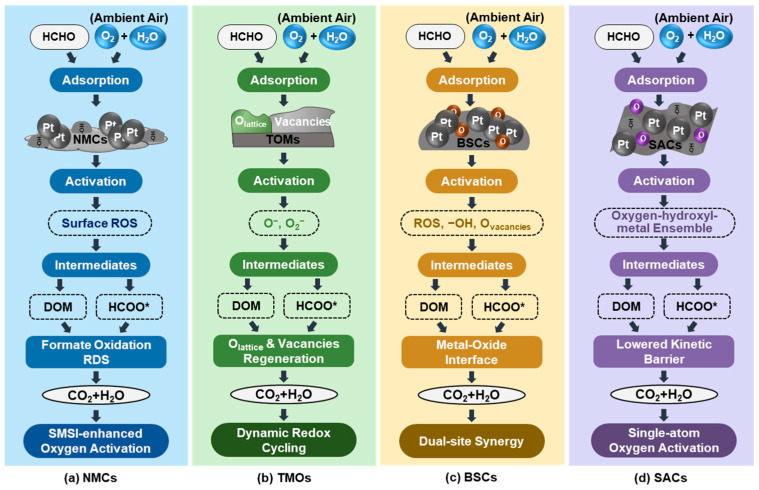
Room-temperature air-only catalytic oxidation pathway of NMCs, TMOs, BSCs, and SACs. Subpanels (**a**–**d**) correspond to NMCs (hydroxyl- and oxygen-assisted pathways), TMOs (dynamic redox cycling mechanisms), BSCs (dual-site synergy mechanisms), and SACs (single-atom oxygen activation mechanism), respectively. Key intermediates and active oxygen species involved in each pathway are indicated, corresponding to the mechanistic descriptions in the main text. The HCOO* represents the formate species.

Formaldehyde (HCHO) catalytic oxidation at ambient temperature generally proceeds through surface-adsorbed intermediates. It is highly sensitive to the nature of active sites, surface oxygen species, hydroxyl groups, and oxygen vacancies. Despite various catalyst systems having been developed, including NMCs, TMOs, BSCs, and SACs, their reaction mechanisms depend on the molecular-level catalytic features. Meanwhile, the overall reaction pathways can be broadly categorized based on dominant active species and observed intermediates across different catalyst systems, as shown in Figure 1.

(1) NMCs: Hydroxyl- and oxygen-assisted pathways

NMCs typically follow hydroxyl- and oxygen-assisted pathways, in which formate species (HCOO*) act as key surface intermediates. HCHO initially adsorbs on surface hydroxyl groups via hydrogen bonding, polarizing the C-H bond and facilitating nucleophilic attack by activated oxygen species. Molecular oxygen is dissociated on metallic noble metal sites into surface ROS, which oxidize adsorbed HCHO into dioxymethylene (DOM) and formate (HCOO*) intermediates (Figure 1a). The deep oxidation of formate to CO_2_ and H_2_O is widely recognized as the rate-determining step (RDS) at room temperature. Strong metal-support interactions (SMSI) enhance oxygen activation, stabilize highly dispersed metal species, and generate oxygen vacancies and surface hydroxyls near noble metal sites, thereby accelerating formate decomposition and enabling complete mineralization.

(2) TMOs: dynamic redox cycling mechanisms

TMOs predominantly operate via dynamic redox cycling mechanisms, where lattice oxygen directly participates in HCHO oxidation, forming DOM and formate intermediates and leaving surface vacancies. These vacancies, along with defect-rich sites, facilitate the activation of gaseous O_2_ and the continuous generation of ROS, including lattice oxygen (O_lattice_), chemisorbed oxygen (e.g., O^−^, O_2_^−^) (Figure 1b), and surface hydroxyl groups, which mediate the subsequent oxidation of intermediates into CO_2_ and H_2_O. Catalytic activity strongly correlates with lattice oxygen mobility, oxygen vacancy concentration, and redox couples (e.g., Mn^3+^/Mn^4+^, Co^3+^/Co^2+^). Defect-engineered TMOs, such as MOF-derived MnO_2_ and δ-MnO_2_, exhibit enhanced low-temperature activity and resistance to intermediate accumulation. Similar defect-driven strategies have been demonstrated in thermally treated zeolitic frameworks, where oxygen vacancies and defect-trapped electrons modify surface electronic structures and govern oxygen activation and VOC interaction at ambient conditions [64].

(3) BSCs: Dual-site synergy mechanisms

BSCs exhibit dual-site cooperative mechanisms, enabling the coexistence and spatial coupling of multiple key active species, including ROS, surface hydroxyls, and oxygen vacancies, under ambient conditions. Typically, noble metal sites facilitate efficient molecular oxygen activation, while oxide components supply abundant hydroxyl groups, oxygen vacancies, and redox-active centers (labeled as oxygen (O) in Figure 1c) for HCHO adsorption and intermediate stabilization. Such cooperative effects simultaneously accelerate HCHO activation, formate oxidation, and active site regeneration, thereby overcoming the kinetic limitations commonly observed in single noble metal or oxide catalysts and resulting in significantly enhanced overall reaction kinetics at ambient temperature.

(4) SACs: Single-atom oxygen activation mechanism

SACs represent a distinct mechanistic regime for ambient formaldehyde oxidation, in which catalytic activity is governed by atomically defined oxygen-hydroxyl-metal ensembles (Figure 1d) rather than metallic clusters, bulk lattice oxygen, or spatially separated synergistic sites. Isolated metal atoms anchored on reducible oxides modulate the local electronic structure, stabilizing adjacent oxygen vacancies and activating lattice hydroxyls (O_lattice_H), thereby enabling continuous oxygen activation and directional oxygen transfer. Unlike NMCs, TMOs, or BSCs, SACs confine adsorption, intermediate conversion, and active-site regeneration within a single atomic-scale reaction unit, which stabilizes key transition states and effectively lowers the kinetic barrier for formate oxidation to CO_2_. Importantly, the superiority of SACs does not arise from introducing new oxygen species but from reorganizing existing oxygen species into a highly efficient atomic-scale reaction architecture, enabling stable and efficient HCHO mineralization at or below room temperature.

Across different catalyst systems, a common kinetic feature emerges: the deep oxidation of formate intermediates is the rate-determining step. Catalysts that can simultaneously provide (i) highly active oxygen species, (ii) abundant surface hydroxyl groups, and (iii) fast oxygen vacancy regeneration exhibit superior low-temperature activity and long-term stability. Therefore, the design of advanced HCHO oxidation catalysts should focus on optimizing oxygen activation pathways, defect engineering, and metal-support interactions, rather than merely increasing surface area or noble metal loading.

#### 4.3.2. Non-Formaldehyde VOCs Oxidation Mechanisms

Compared with formaldehyde, non-formaldehyde VOCs such as aromatic hydrocarbons, aldehydes, and esters exhibit higher molecular stability and more complex oxidation pathways. Oxidation over single-atom and atomic-scale catalysts is generally considered to proceed through radical-mediated pathways involving reactive oxygen species, particularly hydroxyl radicals (·OH). These catalysts exhibit a consistent structural feature: atomically dispersed metal centers (e.g., Pt, Pd, Fe, Ni, Ag) anchored on defect-rich supports, including oxides or heteroatom-doped carbons, form electronically asymmetric coordination environments that simultaneously activate molecular oxygen and interfacial water. This process continuously generates highly reactive hydroxyl radicals (·OH), accompanied by peroxides (O_2_^2−^) or carbon-centered radicals (R·), as evidenced by electron paramagnetic resonance (EPR) [47,51,52], electron spin resonance (ESR) [48], or salicylic acid-impregnated membrane capture coupled with high-performance liquid chromatography (SA-HPLC) [50] in previous studies. In situ diffuse reflectance infrared Fourier transform spectroscopy (DRIFTS) studies [53] further confirmed that the consumption and regeneration of ·OH sustain a self-propagating cycle that drives deep oxidation during non-formaldehyde VOCs oxidation, indicating that ·OH acts as the primary oxidant in the reaction.

**Figure 2:**
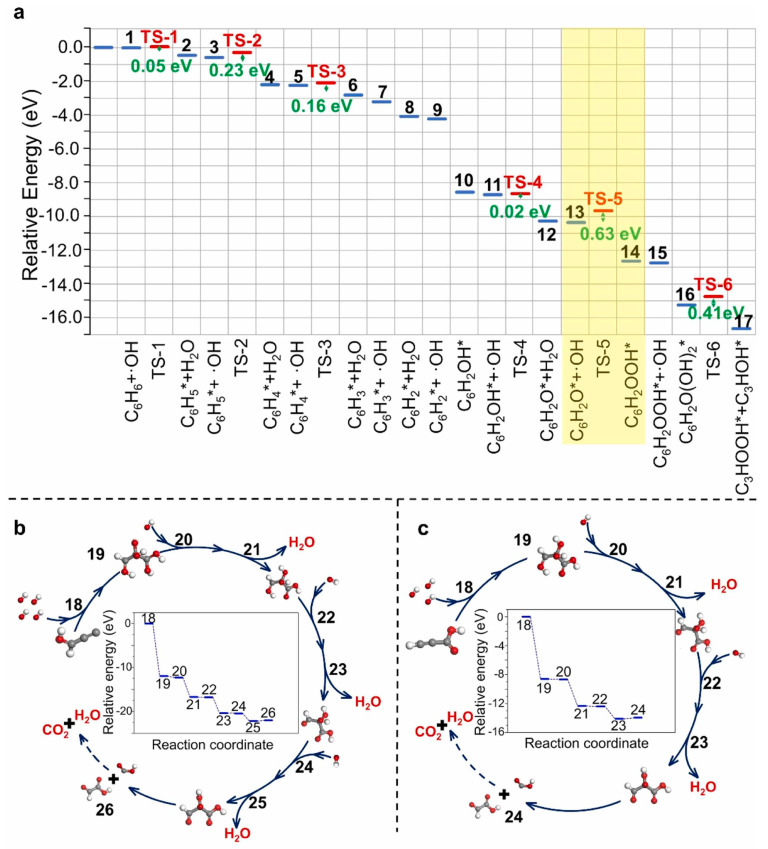
Reaction pathways for room-temperature air-only catalytic oxidation of benzene via ·OH radicals. (**a**) Oxidation pathway of benzene forming C_3_ intermediates; (**b**,**c**) Further oxidation of C_3_HOH* (**b**) and C_3_HOOH* (**c**) intermediates to CO_2_ and H_2_O by ·OH radicals. Key steps in the diagram correspond to the reaction steps described in the main text. The gray, white and red balls represent C, H and O atoms, respectively [50].

**Figure 3:**
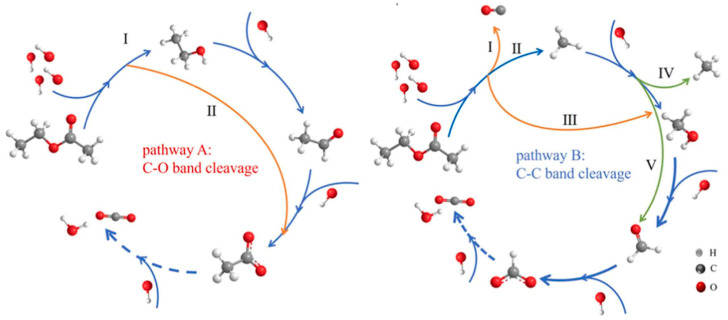
Reaction pathways for room-temperature air-only catalytic oxidation of ethyl acetate via ·OH radicals. Pathway A represents preferential C-O bond cleavage, generating short-chain oxygenated intermediates such as ethanol and acetaldehyde. Pathway B represents C-C bond cleavage, producing intermediates including CH_3_OH and formaldehyde. Both pathways lead to CO_2_ and H_2_O. Key bond cleavages and intermediates in the diagram correspond to the reaction steps described in the main text. The dashed arrows represent complete oxidation, solid arrows indicate each step of the catalytic oxidation process, and different colors of solid arrows correspond to different oxidation mechanisms [54].

For aromatic VOCs, oxidation typically proceeds via stepwise dehydrogenation, hydroxylation, ring-opening, and decarboxylation, with aromatic ring cleavage constituting the rate-determining step, as shown in in Figure 2 [50]. Steps 13–14 correspond to the aromatic ring-opening, representing the key transformation that enables subsequent oxidation of benzene to smaller intermediates and ultimately CO_2_ and H_2_O. The room-temperature air-only catalytic oxidation pathway of benzene to CO_2_ involving the generated ·OH radicals was further supported by density functional theory (DFT) calculations. Oxidation proceeds predominantly via gas–solid interfacial radical chemistry: ·OH radicals first abstract hydrogen atoms from the benzene ring, forming a partially dehydrogenated intermediate; subsequent reaction with ·OH opens the ring, generating smaller C_3_ intermediates; these species then undergo successive hydroxylation, dehydrogenation, and cleavage, ultimately oxidizing to CO_2_ and H_2_O. In contrast, oxygenated VOCs such as ethyl acetate undergo preferential C-O (pathway A in Figure 3) or C-C (pathway B in Figure 3) bond cleavage initiated by ·OH attack, forming short-chain oxygenated intermediates such as ethanol, acetaldehyde, CH_3_OH, and formaldehyde, which are subsequently oxidized to CO_2_ and H_2_O. Pathway A mainly involves sequential C-O bond scission, while Pathway B proceeds through simultaneous C-C and C-O bond cleavage, with partial radicals reacting with surface H and OH, ultimately driving rapid mineralization. The room-temperature air-only catalytic oxidation of ethyl acetate was investigated by in situ Fourier transform infrared (FTIR) analysis (Figure 3) [54]. These oxygenated VOCs generally exhibit lower energy barriers and shorter reaction pathways than aromatic VOCs.

Overall, non-formaldehyde VOC oxidation over atomic-scale catalysts is widely reported to involve hydroxyl-radical-dominated pathways, rapid radical regeneration, and atomic-level oxygen activation, establishing a radical-driven mechanism distinct from that of formaldehyde oxidation. This mechanism circumvents the intrinsic kinetic limitations of surface-bound formate oxidation, which dominate formaldehyde systems, complements traditional kinetic models, and provides a mechanistic basis for the efficient oxidation of structurally stable VOCs under ambient air-only conditions.

## 5. Conclusions and Perspectives

### 5.1. Conclusions

The room-temperature air-only catalytic oxidation represents a green, efficient, and safe approach for VOCs removal, offering notable advantages and broad application potential for indoor air purification. Compared with conventional thermal catalysis or adsorption-based technologies, room-temperature air-only catalytic oxidation can achieve pollutant degradation under ambient conditions without external heating or secondary pollution, thus aligning with the goals of carbon neutrality and sustainable urban development. This review has summarized recent progress of room-temperature air-only catalytic oxidation for typical indoor VOCs, including both formaldehyde and non-formaldehyde species, with an emphasis on catalyst design strategies, thermodynamic and kinetic analyses, and novel reaction mechanisms. The collective understanding of these aspects provides a solid foundation for promoting next-generation catalytic materials and practical air-cleaning devices.

(1) Catalyst systems

NMCs, TMOs, BSCs, and SACs each possess unique merits that determine their applicability in room-temperature air-only catalytic oxidation. NMCs (such as Pt, Pd, and Au) exhibit high intrinsic activity and stability, while TMOs provide tunable redox properties and abundant oxygen vacancies at lower cost. BSCs integrate complementary functionalities between two metallic components, improving oxygen activation and electron transfer efficiency. In particular, SACs show outstanding performance under high-concentration and complex VOC environments due to their atomically precise active sites and interface modulation capabilities, enabling both enhanced catalytic selectivity and long-term durability under realistic indoor air-only conditions.

(2) Non-formaldehyde VOCs removal

For structurally stable VOCs such as benzene, toluene, and esters, constructing synergistic catalytic centers and defect-enriched interfaces has effectively enhanced low-temperature mineralization efficiency. Tailoring the surface electronic structure and optimizing the balance between adsorption and desorption capacities have proven essential for improving deep oxidation activity and maintaining catalyst stability under humidity variations. These developments highlight the importance of combining redox-active components with conductive or porous supports to facilitate the complete mineralization of non-formaldehyde VOCs.

(3) Reaction mechanisms

VOC oxidation reactions are strongly exothermic and thermodynamically spontaneous at ambient conditions, while their kinetics are primarily governed by catalyst structure and active-site configuration. Mechanistic studies have expanded from classical MVK and L-H models to non-classical synergistic pathways characteristic of SACs. Typically, room-temperature air-only catalytic oxidation of VOC involves adsorption of target gas molecules, water, and oxygen species on the catalyst surface, followed by generation of reactive oxygen species, such as ·OH, R·, ·O_2_^−^. These radicals attack the adsorbed VOCs, producing intermediates that are further oxidized to CO_2_ and H_2_O, while the catalyst surface is regenerated. The efficiency of this pathway depends on the nature of the active sites, particularly their electronic structure, which governs ·OH formation.

(4) Thermodynamic and kinetic analyses

The oxidation reactions of numerous VOCs are strongly exothermic and accompanied by substantial entropy increases, confirming thermodynamic spontaneity at room temperature. Kinetic regulation through catalyst design remains critical for improving VOC removal efficiency and CO_2_ mineralization rates. In particular, lowering activation barriers and promoting rapid surface oxygen exchange are central to achieving complete oxidation under ambient, air-only conditions. Combining thermodynamic and kinetic principles will guide the design of catalysts capable of maintaining high activity, selectivity, and stability in real-world indoor environments.

Despite significant advances, several challenges persist for the practical implementation of room-temperature air-only catalytic oxidation in real indoor environments. These include limited complete mineralization capacity of SACs, competitive and degradation mechanisms among multi-component VOCs, and issues regarding catalyst lifetime, regeneration, and cost at the engineering scale.

### 5.2. Perspectives

Future research directions should focus on achieving the comprehensive integration of atomic-level precision, scalable synthesis, multi-pollutant synergy, and intelligent system engineering to accelerate the practical deployment of room-temperature air-only catalytic oxidation technologies for indoor VOCs purification. Addressing these challenges requires cross-disciplinary innovation that bridges chemistry, materials science, artificial intelligence (AI), and environmental engineering.

(1) Dynamic evolution and interface regulation of catalytically active sites

Under realistic reaction conditions, the geometric configuration and electronic states of catalytically active sites undergo continuous dynamic evolution influenced by temperature, humidity, and reactant interactions. These transformations determine catalytic reactivity yet remain challenging to monitor in real time. Future research should prioritize interface engineering and atomically precise structural modulation strategies to stabilize transient active species and optimize oxygen activation. Designing dual-atom catalysts or fully exposed metal cluster catalysts is expected to enhance oxidation depth and durability through multi-metal synergistic effects, particularly under conditions of high humidity and gas hourly space velocity. In parallel, integrating in situ/operando characterization (e.g., synchrotron spectroscopy) with density functional theory and machine learning will enable dynamic visualization of structure-activity relationships and guide the rational design of next-generation catalysts.

(2) Scalable and controllable synthesis of atomically dispersed materials

While SACs and sub-nanometer clusters exhibit exceptional catalytic performance, their large-scale synthesis remains a major bottleneck hindering industrial application. Current synthetic routes, such as atomic layer deposition, electrostatic adsorption, and defect anchoring, often suffer from limitations in yield, reproducibility, and long-term stability. Future work should focus on developing high-throughput, controllable, and cost-effective synthesis strategies capable of maintaining atomic dispersion at gram or kilogram scales, ensuring the practical applicability of room-temperature air-only catalytic oxidation for indoor VOC removal. Combining top-down and bottom-up fabrication approaches with continuous flow synthesis and aerosol-assisted methods may bridge laboratory precision with industrial scalability. Since VOC degradation in indoor environments typically involves low concentrations and large air volumes, it is also important to explore strategies for introducing atomically dispersed active sites onto the pores of structured support materials, thereby maintaining high activity, selectivity, and stability under realistic operating conditions.

(3) Construction of multi-pollutant synergistic degradation models

Indoor environments typically contain complex VOC mixtures where competitive adsorption and reaction intermediates significantly influence catalytic efficiency. Understanding the competitive and cooperative degradation pathways is essential for efficient co-removal of multiple VOCs. Future studies should establish multi-pollutant kinetic and mechanistic models integrating microscopic reaction pathways with macroscopic mass-transfer phenomena. Advanced simulations, such as kinetic Monte Carlo and reactive molecular dynamics, can elucidate the interactions among intermediates and active oxygen species, enabling rational design of catalysts that achieve both high removal efficiency and deep mineralization with minimal byproduct formation.

(4) Multi-scale structure correlation and cross-disciplinary convergence

Bridging the gap between microscopic structural features and macroscopic performance represents a central challenge. AI-assisted materials informatics offers powerful tools to accelerate catalyst discovery through database construction, high-entropy structure analysis, and predictive model validation. Establishing closed-loop frameworks that combine data-driven screening with experimental feedback can enable precise tailoring of catalytic and carbon-capture materials. This approach will facilitate the development of multifunctional catalysts coupling VOC oxidation with CO_2_ capture and conversion, contributing to carbon-neutral indoor environmental management.

(5) System integration and real-environment implementation

To achieve real-world applicability, room-temperature air-only catalytic oxidation technologies must evolve from isolated laboratory studies to integrated systems compatible with ventilation units, air purifiers, and building materials. Real-time monitoring under fluctuating humidity, temperature, and pollutant concentration is essential for optimizing performance and stability. Coupling air-only catalytic oxidation processes with smart control algorithms and self-regenerative functions can enhance energy efficiency and operational lifetime. The integration of these catalytic modules into smart building networks and devices like air conditioners will advance the synergistic development of healthy indoor environments and intelligent city infrastructures.

In summary, the development of room-temperature air-only catalytic oxidation has transitioned from empirical material discovery toward mechanism-guided and AI-assisted atomic-scale design. Through integration of advanced synthesis, dynamic characterization, and intelligent optimization, future research will accelerate the transition from laboratory-scale feasibility to large-scale engineering applications, supporting the sustainable development of next-generation indoor air purification technologies within a global carbon-neutral framework.

## Figures and Tables

**Table 1:** Summary of representative catalysts, reaction conditions, and performance for Room-Temperature Air-only Catalytic Oxidation of HCHO.

Catalyst Type	Catalyst Name	Initial Concentration	Reaction Temperature	Gas Hourly Space Velocity	Reaction Humidity	Removal Efficiency	CO_2_ Mineralization Rate	Ref.
NMCs	Pd/TiO_2_	140 ppm	RT	95,000 h^−1^	40%	100%	-	[20]
	Pt/NiO	200 ppm	RT	5-W fan	-	89%	100%	[21]
	Pd@TS-1	100 ppm	RT	100,000 mL g^−1^h^−1^	35 %	100%	-	[22]
	Pt/Co_3_O_4_	210 ppm	RT	5-W fan	-	91.4%	100%	[29]
	Pt/NiO	200 ppm	RT	5-W fan	50%	90%	100%	[30]
	Pt/SnOx	172 ppm	RT	-	-	87%	100%	[31]
TMOs	MnO_2_/NCNT	100 ppm	30 °C	30,000 mLg^−1^h^−1^	-	≥95%	<20%	[14]
	Fe/δ-MnO_2_	3.35 ppm	23–27 °C	-	40 %	99.4 %	-	[32]
	3D-NiCo_2_O_4_	200 ppm	25 °C	60,000 h^−1^	-	95.3%	100%	[33]
	δ-MnO_2_	22 ppm	30 °C	200,000 mL g^−1^h^−1^	50%	96%	<50%	[34]
	MnO_2_-MOF	0.81 ppm	25 °C	300,000 mL g^−1^h^−1^	50–55%	95%	-	[35]
BSCs	Pt/NiCo_2_O_4_-NF	200 ppm	RT	5-W fan	-	90%	100%	[36]
	Pt/MnO_2_-CF	200 ppm	25 °C	-	-	91%	100%	[37]
	K-Pt/NaY	300 ppm	25 °C	5-W fan	35–51%	98%	100%	[38]
	Au/Co-LDH	200 ppm	RT	5-W fan	-	96.2%	100%	[39]
	Pt/HNaCo_2_O_4_/T_2_	150 ± 5 ppm	RT	5-W fan	50 ± 5%	>96%	100%	[40]
SACs	Pt-MnOOH/MnO_2_	15 ppm	25 °C	30,000 mL g^−1^h^−1^	45%	98.4 %	85.70%	[26]
	Pt/Mn-TiO_2_	100 ppm	15–40 °C	60,000 mL g^−1^h^−1^	50%	100 %	100%	[27]
	Pt_n_/TiO_2_	100 ppm	RT	47,771 mL g^−1^h^−1^	0%	100%	100%	[28]

RT: Room Temperature. CO_2_ Mineralization Rate (%) = C′_out_/(C_in_ − C_out_) × 100%, where C′_out_ denotes the measured gaseous CO_2_ concentration at the reactor outlet after background CO_2_ correction (ppm), C_in_ and C_out_ represent the inlet and outlet HCHO concentration (ppm), respectively. The calculation assumes a complete carbon balance based on HCHO conversion to CO_2_. Initial concentrations reported in the original literature with different units have been converted to ppm for consistency. Space velocities are reported as in the original literature because complete reactor parameters required for conversion to a single metric (e.g., catalyst bed density or bed volume) are not available in most studies. In studies employing a 5-W fan, the reaction chamber volume is typically reported as ~6 L, which represents a common experimental setup and ensures sufficient mixing under room-temperature conditions.

## Data Availability

No new data were created or analyzed in this study.

## Abbreviations

The following abbreviations are used in this manuscript:

VOCs	volatile organic compounds
NMCs	noble metal catalysts
TMOs	transition metal oxides
BSCs	bimetallic synergistic catalysts
LDHs	layered double hydroxides
SACs	single-atom catalysts
WHO	World Health Organization
IAQ	indoor air quality
HCHO	formaldehyde
RT	room temperature
SMSI	strong metal-support interactions
MVK	Mars-van Krevelen
L-H	Langmuir-Hinshelwood
E-R	Eley-Rideal
ROS	reactive oxygen species
DOM	dioxymethylene
RDS	rate-determining step
EPR	electron paramagnetic resonance
ESR	electron spin resonance
SA-HPLC	salicylic acid-impregnated membrane capture coupled with high-performance liquid chromatography
DRIFTS	diffuse reflectance infrared Fourier transform spectroscopy
DFT	density functional theory
FTIR	Fourier transform infrared
AI	artificial intelligence
